# Exploring Primary Care Clinicians’ Views about How Best to Implement a Potential Trial around Point-of-Care Tests for Common Infections in South Africa

**DOI:** 10.3390/diagnostics11112100

**Published:** 2021-11-13

**Authors:** Alice Epps, Charlotte Albury, Oliver Van Hecke

**Affiliations:** Nuffield Department of Primary Care Health Sciences, University of Oxford, Oxford OX2 6GG, UK; charlotte.albury@phc.ox.ac.uk (C.A.); oliver.vanhecke@phc.ox.ac.uk (O.V.H.)

**Keywords:** POCTs, primary care, thematic analysis, prescribing

## Abstract

Optimisation of antibiotic prescribing is critical to combat antimicrobial resistance. Point-of-care tests (POCTs) for common infections could be a valuable tool to achieve this in primary care. Currently, their use has primarily been studied in high-income countries. Trials in low-and-middle-income countries face challenges unique to their setting. This study aims to explore the barriers and facilitators for a future trial of POCTs for common infections in South Africa. Twenty-three primary care clinicians in the Western Cape Metropole were interviewed. Interview transcripts were analysed using thematic analysis. We identified three key themes. These themes focused on clinicians’ views about proposed trial design and novel POCTs, clinicians’ perspectives about trial set-up, and specific trial procedures. Participants were overall positive about the proposed trial and POCTs. Potential issues centred around the limited space and technology available and participant retention to follow-up. Additionally, impact on clinic workload was an important consideration. These insights will be invaluable in informing the design of a feasibility trial of POCTs in this setting.

## 1. Introduction

Overuse of antibiotics is a key driver of antimicrobial resistance (AMR). Antibiotic use is associated with increased levels of resistance at the level of the individual [1] and the community [2]. In addition, resistant infections are associated with more severe symptoms and increased rates of reconsultation [3]. Hence, optimising antibiotic prescribing is a crucial step in reducing the impact of AMR. Respiratory tract infections (RTIs) are the most common reason for antibiotic prescribing in primary care [4,5,6]. Primary care clinicians may prescribe antibiotics “just in case” if facing uncertainty [7], and reducing clinical uncertainty may reduce such prescribing [7]. POCTs for acute infections may help to do this. Recent evidence shows POCTs can reduce antibiotic prescribing for acute infections in primary care [8,9,10]. South Africa has a particularly high level of antibiotic use per capita, compared to many other countries [11], and antibiotic use in South Africa increased the most of all the BRICS nations (Brazil, Russia, India, China and South Africa) between 2000 and 2010 [12]. There is limited published research regarding antibiotic prescribing in South African ambulatory care, particularly in the public sector. In a rare point prevalence study across eight primary care facilities in the Cape Town Metro District, acute cough was the commonest presenting symptom [13]. However, broad-spectrum antibiotics were commonly prescribed, and only 1 in 3 antibiotic prescriptions were guideline-congruent [13]. Therefore, methods to optimise antibiotic prescribing are much needed here. Previous work by van Hecke et al. explored primary care clinicians’ views of POCTs for common infection syndromes (acute cough, urinary tract infections) in primary care clinics in the Western Cape Metro region of South Africa [14]. They found that clinicians saw the potential for POCTs to reduce antibiotic overprescribing.

Although clinicians’ views about the introduction of POCTs have been explored [14], there is little evidence around what may facilitate or hinder the implementation of a future POCT trial in these settings. Therefore, in this study, we aim to identify specific barriers and facilitators to a future trial of POCTs in this setting using the qualitative data that were collected but not analysed in the original study. Identifying these barriers and facilitators is important because clinical trials in resource-poor settings experience context-specific limitations that are essential to address before implementation [9].

## 2. Methods

### 2.1. Participants

Participants were nurses and doctors (family physicians and non-specialist doctors) responsible for making antibiotic-prescribing decisions for patients with common infection syndromes in the Western Cape Metropole, South Africa.

A convenience sampling approach was used. In this approach, investigators enrol eligible subjects who are available and accessible [15]. Participants were recruited through several ways: advertising the study at a nurse managers’ workshop at the University of the Western Cape and the Cape Town Family Physician Forum; personal contacts at the Department of Family Medicine, University of Cape Town; and snowballing (using participants’ contacts to recruit further participants). Participants were informed of the purpose of the study during the workshops/fora and reminded of this in the participant information leaflet as well as in the prelude of the topic guide.

### 2.2. Interviews

O.V.H., a postdoctoral clinical researcher, conducted semi-structured interviews. Interviews were conducted in English and followed a topic guide (see Appendix A) that had been piloted. Questions explored clinicians’ perceptions of a potential future trial of a POCT for common infection syndromes (e.g., acute cough). Participants were asked to comment on specific aspects of the trial, including recruitment, randomisation, and patient follow-up, to highlight participants’ perceptions of barriers and facilitators to each aspect. Written consent was gained before interviews. Interviews were audio-recorded and transcribed verbatim.

### 2.3. Analysis

Interview transcripts were analysed by the lead author using thematic analysis [16] aided by NVivo 12 software (QSR International, Burlington, MA). The lead author used line-by-line coding for the first 10 transcripts. These codes were then organised into a coding framework that all authors discussed and refined. The lead author then coded the remaining transcripts using this coding framework. We took an interpretivist approach, aiming to elicit and understand the views of clinicians regarding a future trial of POCTs. The study used an inductive approach to coding, such that the coding was driven by the clinicians’ responses. To ensure the trustworthiness of the results, we kept an audit trail and had regular peer debriefings. The themes are descriptive, summarising clinicians’ views and perspectives on what may facilitator or hinder the implementation of a future POCT trial.

### 2.4. Reflexivity

The influence of the researcher on qualitative research is inevitable, so, in line with best practice in the field of applied qualitative health research, we used a process called reflexivity. Reflexivity involves remaining cognisant of one’s background and beliefs and making these clear to the reader. We were aware of our backgrounds and the possible effects on the data, and A.E. kept a reflexivity journal throughout the process. A.E. is a medical student with an interest in AMR and POCTs. She has no experience in qualitative research or working abroad and has limited experience in clinical medical practice. O.V.H. is a principal investigator of published qualitative studies and has partaken in formal training and teaching on qualitative research methods. The researchers were aware of the possible biases introduced by O.V.H., having trained in South Africa, including the choice of research location. C.A. is a non-clinical qualitative methodologist who actively challenged any clinical biases. All researchers challenged each other’s biases to ensure the results were grounded in the data.

## 3. Results

In total, 23 primary care clinicians working at 13 different clinics were interviewed. Of these, 14 were female. There were 10 specialist family physicians, 8 nurse practitioners, 4 medical officers (non-specialist doctors) and 1 Family Medicine trainee registrar. The majority of these (21/23) had at least 10 years in practice. The average age of participants was 42 years. For further details of participants, see Appendix B. The mean duration was 38 min (range 22 to 59 min). 

The main themes and subthemes are summarised in Table 1. Each theme is illustrated with quotations from participants, identified by participant code (Appendix B).

### 3.1. Theme 1. Clinicians’ Views about Proposed Trial Design and POCTs

Most clinicians reported feeling optimistic about the proposed trial and the introduction of novel POCTs in clinics. Many agreed that the trial seemed achievable. Clinicians perceived many benefits of novel POCTs. For example, many stated that they could aid in the diagnosis of respiratory illnesses and urinary tract infections. Others thought POCTs could save money and would be beneficial for antimicrobial stewardship efforts.

“*You know something like this could make a big change in a clinic […] like this. You know, money-saving and treating [infections] appropriately*.”

(P12)

As well as the specific barriers discussed below, more general considerations for the trial were also identified. A few clinicians stated that clinics were also facing other challenges, for example, high levels of HIV and TB in the population, which increased their workload.

“*We have got such [high] HIV and TB problems that we also need to focus on*.”

(P1)

Participants’ views of POCTs may have been influenced by previous experiences with other POCTs. For example, a POCT for TB was available in some clinics, which saved time compared to sending samples to the lab. The frequent use of urine dipsticks meant clinicians could identify possible issues with such POCTs. For example, urine samples can be contaminated. Others had a limited understanding of POCTs due to lack of experience but were nonetheless interested in “*what these POCTs are and what they offer.*” (P5)

### 3.2. Theme 2. Clinicians’ Perspectives about Trial Set-Up

Clinicians expressed a range of views regarding the inclusion of trial sites. A key factor was the size of clinics. Many noted that larger clinics were more commonly chosen for trials, while smaller clinics were “*neglected by researchers*” (P3). Some clinicians supported choosing smaller clinics for the trial as they are eager to be involved in research, have “*more energy*” (P3) and are less “*research-fatigued*” (P23). On the other hand, many clinicians believed it was better to choose larger clinics to make it easier to recruit more patients. Yet other clinicians recommended choosing clinics of varying sizes to investigate the efficacy of POCTs in each setting.

“*I think [you should] take a 24 h [facility], a small facility like this and then […] in between facilities. Between the small and the big ones. Then you will […] see how [POCTs are] actually going to work for the small, medium and large [facilities]*.”

(P7)

Other important factors to consider were the number of other studies happening at clinics and patient demographics. Clinicians noted that clinics with trials already happening might not be able to accept another trial.

Many clinicians identified available clinic space as an important factor in trial set-up. The available space varies considerably between clinics. For example, some clinicians expressed concern about the lack of a safe place to store the POCT machine when not in use.

However, other clinicians perceived space would not hinder the trial. Some noted that they had managed to find space for previous trials, so they should be able to do so again. One way this had been done was by installing mobile clinics outside the facility for the trial. Furthermore, the clinics already have rooms for samples to be taken (“injection rooms”) in which the POCT machine could go. Alternatively, all patients initially go to the triage or “prep room”. After the consultation, the clinician may ask them to return here for further investigations. This room was identified by many clinicians as a suitable place for the POCT machine.

“*Because that’s where we send everyone for […] special [investigations], like [urine] dipsticks or [rapid] HIV [tests], we send them to the prep [room].*”

(P12)

Some clinicians raised concerns regarding the impact of the trial on the workload of the clinics, stating that the trial should not burden already over-stretched staff. However, many responded positively to the fact that the workload would hopefully be shouldered by researchers and not passed on to clinicians or other staff.

“*You just mentioned there will be a tester […], so less time consuming for us. Definitely that will be a plus for the trial.*”

(P21)

Some clinicians were also concerned about the effect of POCTs on patient workflow. However, other clinicians reported the trial would likely not be overly disruptive as the new POCT could fit into existing workflows within the clinics, for example, among other tests taking place in the triage room.

“*The person is going to wait [in the triage room] anyway, because they’re getting their blood pressure done […] they’re not going to immediately go and see a clinician anyway.*”

(P16)

### 3.3. Theme 3. Clinicians’ Perspectives on Trial Procedures

Clinicians reported there would not be any issues with patient recruitment to the trial as the infection syndromes concerned are very common and the clinics see many patients every day. Furthermore, patients are used to participating in trials, which clinicians expected to facilitate recruitment.

“*The community members are already used to the ideas of [being] participants […]. They have been there, done that.*”

(P3)

Participants agreed that the inconsistent internet connectivity would mean that electronic patient randomisation would prove difficult. However, clinicians suggested other ways of randomising patients, including one suggestion of using a random number generator on the researcher’s smartphone. As there would be a limited number of POCT machines available for clinics, the potential trial would involve clinics using a POCT machine every alternate week. Some participants did not have any concerns regarding this approach.

“*I can’t see a big issue particularly with that […], it is a big enough clinic that there would be enough patients to be a random sample.*”

(P5)

However, some participants reported concerns regarding this approach. These mainly focused on factors that may differ week to week and could not be controlled for, for example, changes in clinicians working in the clinics. Another issue regarding randomisation was managing patients’ expectations and explaining why some patients may get a POCT while others would not.

“*It’s just the patient expectations […], they will be coming thinking [they are] getting a test and then they [are] not.*”

(P9)

Participants were wary that because medical records were paper-based, it could lead to challenges such as missing or misfiled notes, which could be a significant hurdle to overcome.

“*It can be very difficult to find [the patient file] if it was not put in the right place or not filed at all.*”

(P3)

Some clinicians identified ways to overcome this issue, including keeping trial notes separate from patient notes so that they do not get lost or keeping a digital copy of notes.

“*I would suggest [trying] to make some copies of [trial notes] […] or otherwise the [record] just literally goes missing.*”

(P4)

Another issue is that “*record keeping in primary care is notoriously poor because of time pressures*” (P23). Hence, patient notes are often limited or of poor quality. While this may be a barrier to the trial, one clinician reported that a current trial has its own clinician to take the patients’ history to ensure all necessary information is collected for the trial. Some clinicians also recommended using a checklist for trial notes (or case report form) to ensure all the necessary information is gathered. However, they noted that this should be concise to reduce the workload for clinicians.

The proposed trial would also involve the follow-up of patients in the community. Clinicians described varying follow-up procedures in their clinics. While many clinicians reported that there was no formal clinic for the follow-up of acute patients at their facility, many clinicians stated it was possible to book an appointment to follow-up a patient.

Others said they would give a patient a date to return for follow-up, although not all patients return, particularly if they are feeling better.

“*Patients that I am really worried about, I would rather just give them a date, then I write my name on it and I tell them you come back to me.*”

(P6)

One of the barriers perceived by clinicians to follow-up in the trial was that in some areas, the populations are very mobile. In addition, in the proposed trial, SMS would be used to ask patients to return for follow-up. However, clinicians noted that some patients do not have a mobile phone and many of those who do change their numbers frequently.

“*A lot of [patients] change their cell phone numbers every three or four months.*”

(P9)

On the other hand, many clinicians identified possible facilitators to follow up in a trial and believed it would be possible to get patients to return. For example, to overcome the above issues with mobile phones, the participants suggested also recording a relative’s phone number. Furthermore, previous trials have used SMS for follow-up, and many clinicians believed it would be effective. Alternatively, clinicians suggested sending community care workers to find patients, as is currently done in some clinics.

“*So if we want someone, there is a pamphlet where it says Mrs. […] you are required to come to the clinic and then our community care workers will drop at the house and then the patient comes.*”

(P10)

Offering an incentive may also facilitate follow-up. Clinicians reported that other trials have offered either financial or non-financial incentives to patients, and those patients tended to return for follow-up if offered an incentive.

“*They just expect to be treated well when they come, so it is kind of like incentives, we will go and, we will fast-track their medication [when they attend clinic].*”

(P3)

## 4. Discussion

Clinicians were enthusiastic about the proposed trial and the possible implementation of new POCTs in their clinics. However, the clinicians also identified possible barriers. These were the limited space and technology available in clinics, concerns about disruption to patient workflows, and difficulties contacting patients for follow-up.

In line with our findings, previous clinical trials in South Africa have highlighted specific challenges to conducting trials in this setting, including limited space [17] and issues with randomisation [18]. For example, trial evaluation of a group intervention for diabetes in South African primary care found limited space available for the intervention to be a key issue [17], which was also identified by clinicians in this study. Botes et al. [17] report that space was a particular issue as facility managers did not prioritise diabetes, instead prioritising HIV and AIDS. Therefore, the views of facility managers regarding the relative importance of AMR could determine the extent to which limited space hinders a future POCT trial in this setting. A priori randomisation was used by Stassen et al. [18] but was identified as one of the factors which may have limited recruitment in the trial as it caused confusion amongst clinicians.

In relation to the impact of POCTs on patient workflow, previous studies also found disruption to workflows to be a barrier to the implementation of POCTs [19,20,21]. Our participants agreed with previous research on offering a financial incentive to increase participant retention, both in HICs [22,23] and in South Africa [24]. However, a Cochrane review found no evidence for the efficacy of offering non-financial incentives to increase follow-up [22].

Despite some barriers to the use of SMS to aid in patient follow-up being identified by clinicians in this study, existing evidence supports the use of SMS as an effective way to increase patient follow-up in trials or attendance at healthcare appointments, both in South Africa [25,26] and in other LMICs [27,28].

We interviewed frontline clinicians currently working in primary care as they are well-placed to identify possible barriers to integrating a trial into current practice [29]. We coded the interviews inductively, meaning the themes were driven by the data [16], and so could pick up barriers and facilitators thought to be important by clinicians. However, the data obtained will inevitably have been shaped by the interview questions chosen by researchers and our own interpretation during analysis. To mitigate these factors, we employed the practice of reflexivity and peer discussion to ensure we remained aware of our own biases and that the results were grounded in the data.

Due to time restrictions, convenience sampling was used. This may have caused sampling bias, favouring clinicians who had had previous positive experiences of trials. Indeed, many clinicians had previously participated in trials and were able to offer valuable insights from these experiences. However, these views were balanced with those of other clinicians with no previous experience in clinical trials. We did not interview patients or non-clinical members of the primary care team, which may have identified further potential barriers not noted by clinicians.

The interviews were conducted in early 2018, so some factors may have changed in this time, for example, available technology and, particularly, changes to healthcare provision due to the SARS-CoV-2 pandemic. Furthermore, the study was conducted in one region in South Africa, so it is difficult to determine the transferability of these results to other areas or countries.

Future research should aim to implement these findings in a feasibility trial of POCTs in this setting. The size of clinics must be considered when selecting trial sites for such a trial, although the use of mobile units could enable smaller clinics to participate. Inconsistent internet access means that randomisation would need to be conducted differently compared to settings that are more affluent. Minimising disruption to patient workflows will be important when planning a future trial, but, as clinicians in this study reported, this could be achieved by fitting the POCT within existing processes. Several methods may be required to ensure sufficient retention rates; these could include SMS reminders, offering small financial incentives, and utilising community care workers.

## 5. Conclusions

This study presents a series of important contextual influences that a future trial of POCTs in South African primary care would need to consider. These include unique barriers to follow up, limitations regarding infrastructure, and low capacity to cope with changes to volume or patterns of work. 

## Figures and Tables

**Table 1:** Main themes and subthemes.

**Theme 1. Clinicians’ views about proposed trial design and POCTs**
1.1 Optimism for proposed trial design and novel POCTs 1.2 Considerations for trial design 1.3 Experiences of other trials 1.4 Previous experiences of POCTs influence clinicians’ perceptions of new POCTs
**Theme 2. Clinicians’ perspectives about trial set-up**
2.1 Differing opinions about inclusion of trial sites (clinics) 2.2 Available space varies and may be either a barrier or facilitator of trial 2.3 POCTs in patient flow
**Theme 3. Clinicians’ perspectives on trial procedures**
3.1 Clinicians identified many ways to recruit patients and few barriers to recruitment 3.2 Randomisation of patients to trial 3.3. Process of sample collection and results 3.4 Available technology at clinic sites is limited so patient notes are paper-based 3.5 Follow-up procedure varies between clinics 3.6 Barriers to follow-up in trial 3.7 Facilitators to follow-up, and suggestions to improve follow-up in trial

## Data Availability

Data are available on reasonable request.

## Appendix A. Interview Topic Guide

This is the extract of the full topic guide that is relevant to my research question. Parts of the interviews corresponding to the rest of the topic guide have previously been analysed with regard to a different research question.

Our team at Oxford are planning a large trial to find out how and where such a POCT would work best in day hospitals/clinics such as this one. The aim of the trial would be to evaluate the clinical and cost effectiveness of POCTs in routine community care for two infection syndromes (acute cough, urinary tract infection) in the Western Cape.

Very briefly, the idea for the trial would be that eligible patients would receive their clinical care in the usual way at day hospitals. Clinicians (doctors and nurses) responsible for their care will then raise the study with them and refer them to the on-site study team if they are interested in considering study participation. The study team member will obtain consent from the patient, record clinical information and any planned follow-up, and take blood and microbiological samples from the participants. As somebody on the ground, working in the day hospitals, I want to ask your advice about what would be the best way to go about doing this study.

1. Firstly, what do you think of the idea of the trial?
  - Prompt—Do you think such a trial is achievable?
2. How do you think we should organise the recruitment of patients?
  - Prompt—How should we select day hospitals for recruitment? Should this be based on geography, rates of antibiotic prescribing, size of the clinics? Where should we recruit patients?
3. What would be a good way to randomise patients to the study?
  - Prompt—For example, a computer system (not a doctor) will decide which patients will have POCTs before they are given treatment. How would such a system work in the clinic? Can we do this online or through a smartphone? Is there a private office available?
4. How do you think we could organise POC testing in the day hospitals?
  - Prompt—How many machines are needed? Where can the POCT be placed safely? How can we avoid it being used when patients are not being randomised to the POCT?
5. How can we ensure that all the clinical information for the trial is properly recorded?
  - Prompt—Is there a system to compare and check patient details?
6. What would be the best way to follow-up patients?
  - Prompt—How often are patients followed-up? Is there a separate clinic for follow-up patients?
7. Patients will also require samples to be collected during the trial. How can we ensure that the correct samples are sent to a special research laboratory in Cape Town?
  - Prompt—What happens to samples that are taken in the clinic? Where are they sent? How often are the collections?

Closing question

Is there anything else that I have not asked but that you would like to tell me? Thank you for taking the time today to help with our research.

## Appendix B. Participant Characteristics

**Table A1:** Summary of participant characteristics.

Participant	Site	Age	Gender	Race	Position	Years in Practice (*)
01	Clinic A	44	Male	White South African	FP	13 (7 since specialisation)
02	Clinic B	48	Male	Mixed race	FP	24 (16)
03	Clinic C	54	Female	Black African	FP	28 (20)
04	Clinic D	32	Male	Mixed race	FP	10 (2)
05	Clinic E	43	Male	White South African	FM SpR	12 (7)
06	Clinic F	31	Female	Mixed race	NP	7 (3)
07	Clinic F	55	Female	Mixed race	NP	39 (20)
08	Clinic F	47	Female	Mixed race	NP	23 (10)
09	Clinic F	35	Female	White South African	MO	11 (0)
10	Clinic F	31	Female	Black African	NP	7 (0)
11	Clinic F	49	Female	Mixed race	NP	28 (15)
12	Clinic G	33	Female	White South African	FP	10 (3)
13	Clinic H	37	Female	White South African	FP	14 (4)
14	Clinic E	39	Male	Mixed race	MO	16 (2)
15	Clinic I	39	Female	Mixed race/coloured	FP	15 (4)
16	Clinic F	46	Male	White South African	FP	21 (6)
17	Clinic J	42	Male	Mixed race/coloured	NP	18 (11)
18	Clinic J	38	Female	Black African	NP	10 (10)
19	Clinic J	53	Female	White South African	NP	33 (20)
20	Clinic K	49	Female	White South African	FP	26 (10)
21	Clinic L	36	Male	Black African	MO	13 (0)
22	Clinic G	41	Male	Black African	MO	15 (0)
23	Clinic M	48	Female	White South African	FP	24 (17)

* Years since specialisation or prescribing nurse. FP = specialist family physician (GP); FM SpR = Family Medicine trainee registrar; NP = nurse practitioner; MO = medical officer (non-specialist doctor).
